# Pigmented Structural Color Actuators Fueled by Near-Infrared Light

**DOI:** 10.1021/acsami.2c03392

**Published:** 2022-04-22

**Authors:** Pei Zhang, Michael G. Debije, Laurens T. de Haan, Albert P. H. J. Schenning

**Affiliations:** † Stimuli-Responsive Functional Materials and Devices, Department of Chemical Engineering and Chemistry, Eindhoven University of Technology, P.O. Box 513, 5600 MB Eindhoven, The Netherlands; ‡ SCNU-TUE Joint Lab of Device Integrated Responsive Materials (DIRM), National Center for International Research on Green Optoelectronics, South China Normal University, Guangzhou 510006, P. R. China

**Keywords:** light-responsive polymers, soft actuators, structural color, thermochromic materials, liquid crystal elastomers

## Abstract

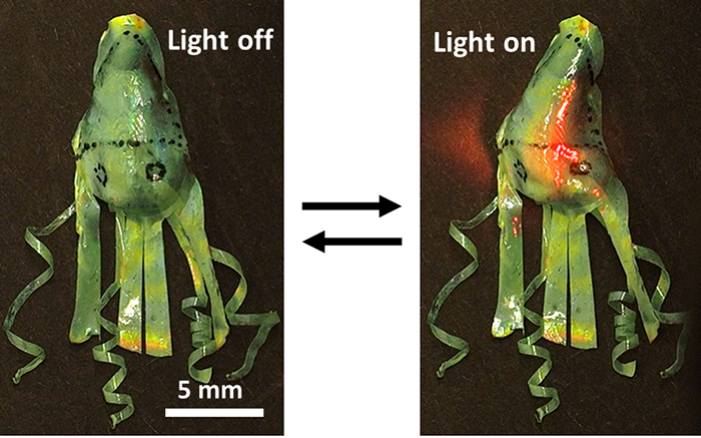

Cuttlefish can modify their body shape and both their pigmentary and structural colors for protection. This adaptability has inspired the development of appearance-changing polymers such as structural color actuators, although in most cases, the original shape has been confined to being flat, and pigmented structural color actuators have not yet been reported. Here, we have successfully created a pigmented structural color actuator using a cholesteric liquid crystal elastomer with a lower actuation temperature where both actuation and coloration (structural and pigmental) are tunable with temperature and NIR light. The shape, structural color, and absorption of the NIR-absorbing dye pigment of the actuator all change with temperature. Light can be used to trigger local in-plane bending actuation in flat films and local shape changes in a variety of 3D-shaped objects. A cuttlefish mimic that can sense light and respond by locally changing its appearance was also made to demonstrate the potential of pigmented structural color actuators for signaling and camouflage in soft robotics.

## 1 Introduction

Sensing and adaptation are important survival traits for organisms. For example, cephalopods adapt both their body shape and color for camouflage: the mimic octopus can rearrange the shape of its entire body to appear as another species, such as a flatfish or a banded sea snake, in order to deceive predators.^1^ Cephalopods rely on cooperativity of both pigmentary and structural color elements distributed throughout their skin to access their diverse range of colors.^2^ Pigmentary color originates from wavelength-selective absorption of light, for example, by dye molecules. Structural color derives from the physical interactions between periodic nanostructures and light.^3,4^ In addition to cephalopods, there are other organisms that combine structural color with pigmental color: buttercup petals have both carotenoid pigments for yellowness and thin films for reflection.^5^ The shape and color adaptions found in nature have inspired the fabrication of soft actuators with integrated color changes in the lab^6^ as such materials can be adapted for use in soft robotics for signaling, camouflage, and temperature regulation.

Stimuli-responsive structural color actuators based on cholesteric liquid crystals (CLCs),^7−18^ cellulose nanocrystals,^19−23^ hydrogels,^24−27^ and opal/inverse opal-structured polymers^28−37^ have been reported. Light has emerged as the most promising stimulus for structural color actuators as it allows wireless and remote activation with spatiotemporally selective capabilities.^38,39^ For example, near-infrared (NIR, 0.76–1.5 μm^40^)-light-responsive bending and color changes can be achieved using a bilayer hydrogel with an inverse opal scaffold or a cellulose nanocrystal/polyurethane bilayer.^21,24^ However, achieving more complex color and shape changes using these materials remains challenging.

CLCs form helical photonic structures that reflect circularly polarized light with the same handedness as the helical structure and are a good candidate to make actuators with synergistic shape and structural color changes.^13,16,41^ A light-responsive structural color actuator that changes color while bending was obtained based on cholesteric liquid crystal elastomers (CLCEs),^11^ but the deformation was confined to bending. Previously, we reported a 4D CLCE actuator with temperature-responsive shape, structural color, and hyper-reflectivity changes. However, high temperatures (>100 °C) were required to drive actuation. We now report an NIR light-driven 4D pigmented structural color actuator based on CLCEs that shows complex deformations. To fabricate a material mimicking the skin of cuttlefish with both structural and pigmental color changes, a CLCE mixture was engineered to achieve temperature-sensitive actuation suitable for light triggering by adding a photothermal NIR dye. The structural color actuators prepared show reversible structural and pigmental color and dimensional changes when exposed to NIR light or temperature variations. A 3D cuttlefish model is assembled from different pigmented structural color actuators: when this 3D cuttlefish is locally exposed to light, it modifies its body shape and color immediately.

## 2 Results and Discussion

### 2.1 Preparation of the Pigmented Structural Color Actuators

**Figure 1:**
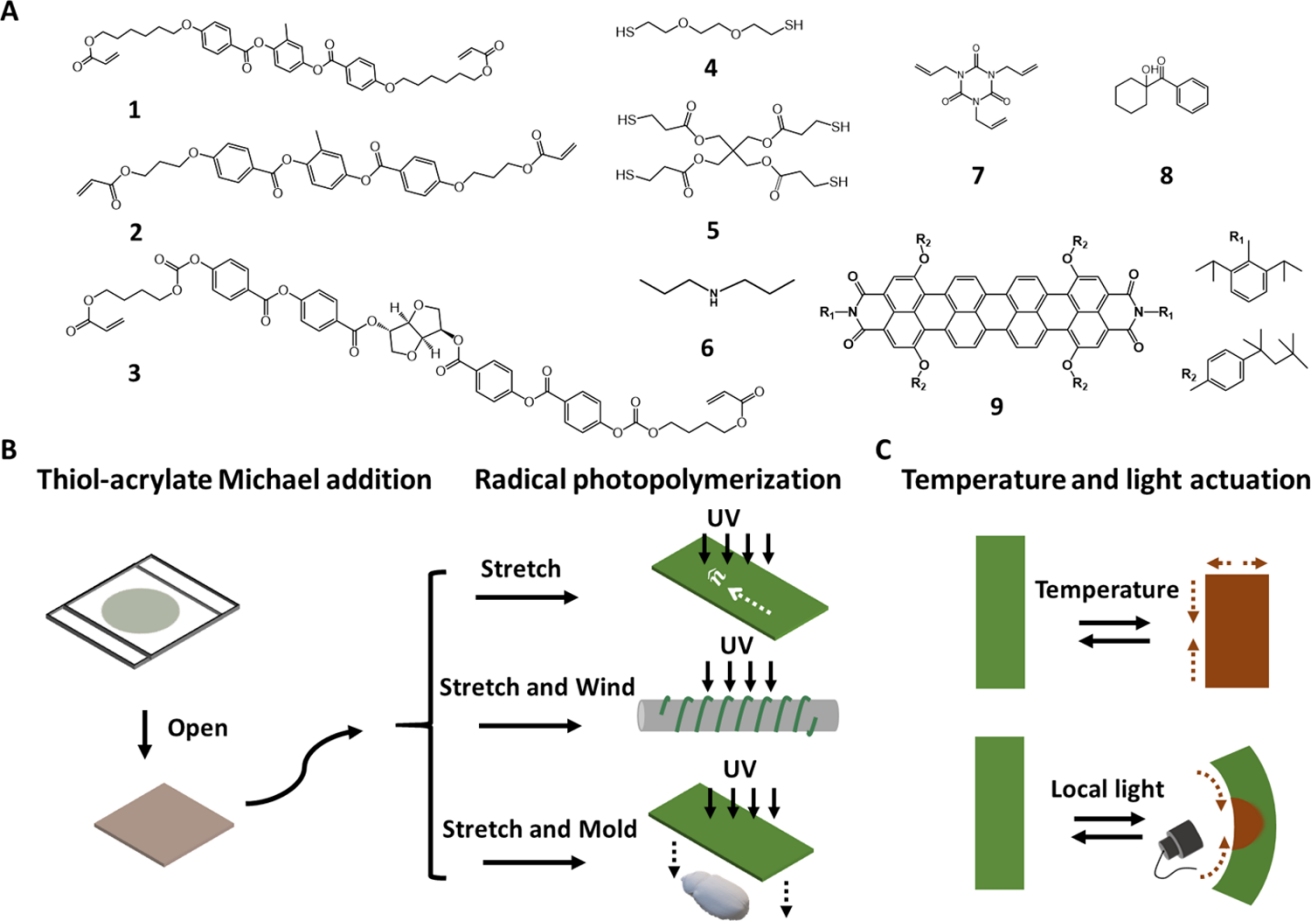
(A) Chemical structures of the molecules used in this study and (B) process to prepare the CLCE films using the two-step crosslinking procedure. (C) Schematic depicting the temperature- and IR-light-fueled actuation.

To program the CLCE film with a lower temperature actuation threshold, we employ a two-stage thiol-acrylate/thiol-ene reaction based on an LC mixture reported earlier by Ware and co-workers.^42^ The compounds for preparing the CLCE are displayed in Figure 1A. The ratio among functional groups was kept as thiol:acrylate:vinyl groups = 1:0.8:0.2, so that after the first-step cross-linking via the thiol-acrylate Michael addition, there are 20% excess thiol groups left, which then reacted with the vinyl groups in the second cross-linking via thiol-ene photopolymerization. Second, tetrathiol **5** acts as a cross-linker for the first-step cross-linking, and the ratio between dithiol **4** and tetrathiol **5** was chosen to ensure 25 mol % thiols coming from tetrathiol, therefore forming a proper cross-linking density after the first cross-linking.^41^ The reactive chiral compound **3** was added to create the cholesteric liquid crystal phase. Then, 0.07 wt % of the photothermal dye IR 788^43^ (**9**) was added as a pigment to the mixture to achieve an IR-light-responsive actuator. The DSC curve of the monomer mixture without the catalyst **6** shows crystallization at 18 °C and a melting point at 65 °C (Figure S1A). The films after first cross-linking via thiol-acrylate Michael addition show a glass transition temperature (*T*_g_) at −20 °C (Figure S1B). However, no obvious isotropic transition was observed. Tensile tests were performed to study the mechanical properties of this CLCE film, which after first cross-linking can be elongated up to 400% before breakage (Figure S2), meaning that there is considerable freedom to program the film before applying the second radical photopolymerization step between the remaining thiol groups and the vinyl groups from cross-linker **7**. The free-standing CLCE film after first cross-linking was uniaxially stretched to a strain of 90% and photo-polymerized at room temperature to fix the shape and color of the film. After this second cross-linking, the *T*_g_ increased to −8 °C (Figure S1B). After fully cross-linking, the CLCE film can be elongated to 120% before it fails (Figure S2), and Young’s modulus of the CLCE film increases dramatically (from 0.073 to 1.85 MPa) compared to after the first cross-linking, attributed to the increasing of cross-link density of the CLCE film.

**Figure 2:**
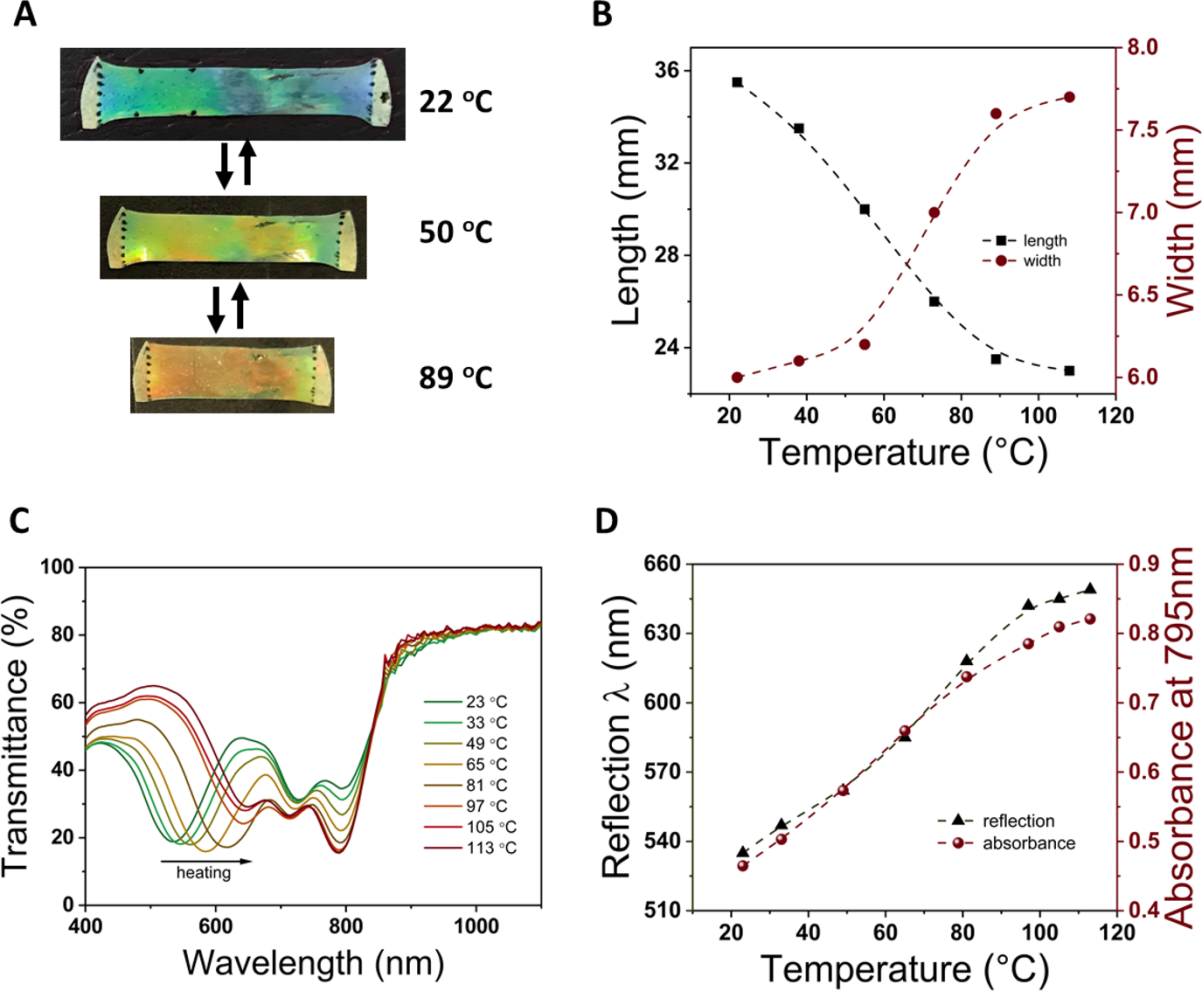
(A) Photographs of the CLCE film doped with IR 788 at different temperatures. (B) Length and width of the film as a function of temperature (dashed lines are plotted to guide the eye). (C) Transmittance spectra of the dye-doped CLCE film measured at different temperatures upon heating. (D) Reflection wavelength (λ) and absorbance at 795 nm as a function of temperature upon heating (dashed lines are plotted to guide the eye).

The resulting CLCE film is colored green at 22 °C (Figure 2A), a combination of the reflection by the cholesteric structure and absorption of light by the embedded dye. When heated to 89 °C, the film becomes red. The length of the film strip decreased by 35.2% (35.5 to 23 mm) with a width increase of 28.3% (6 to 7.7 mm) upon heating from 21 to 100 °C (Figure 2B). The actuation temperature, which is defined as the temperature halfway between the starting and ending temperatures of the most dramatic length change on heating, is found to be 60 °C, which is lower than in the previously reported system (100 °C).^41^ When heated from 23 to 113 °C, the reflection wavelength redshifts from 535 to 650 nm (Figure 2C,D); this shift is reversible upon cooling (Figure S3A and Video S1). Two absorption peaks of the photothermal dye **9** are observed at 725 and 795 nm with a higher intensity of short wavelength band (lower transmittance). The absorption bands have been previously assigned to H-type aggregates in which the dyes are stacked on top of each other.^44^ Remarkably, upon heating, the ratio between these two absorption peaks changes and at 113 °C, the highest absorbance is found at 795 nm with a shoulder at 725 nm (Figure 2C). Such a spectrum is typical for molecular dissolved IR 788 dyes. The absorbance at 795 nm shows an increase from 0.46 to 0.82 when heating from 23 to 113 °C (Figure 2D), and the shift is fully reversible upon cooling (Figure S3A). The spectral changes are likely due to solubility changes of the dye in the CLCE with temperature, with solubility decreasing at lower temperatures causing aggregation and with increasing solubility at higher temperatures resulting in de-aggregation.^44−46^

The alignment of the dye in the film was measured with light polarized both parallel and perpendicular to the stretching direction of the film. The thermochromic changes for both polarizations are the same when the film is heated from 22 to 113 °C (Figure S3B), implying that the absorption behavior of the dye is not dependent on the polarization of the light, and thus there is no dye alignment along the stretching direction.

### 2.2 Unraveling the Temperature-Responsive Structural and Pigmental Colors

**Figure 3:**
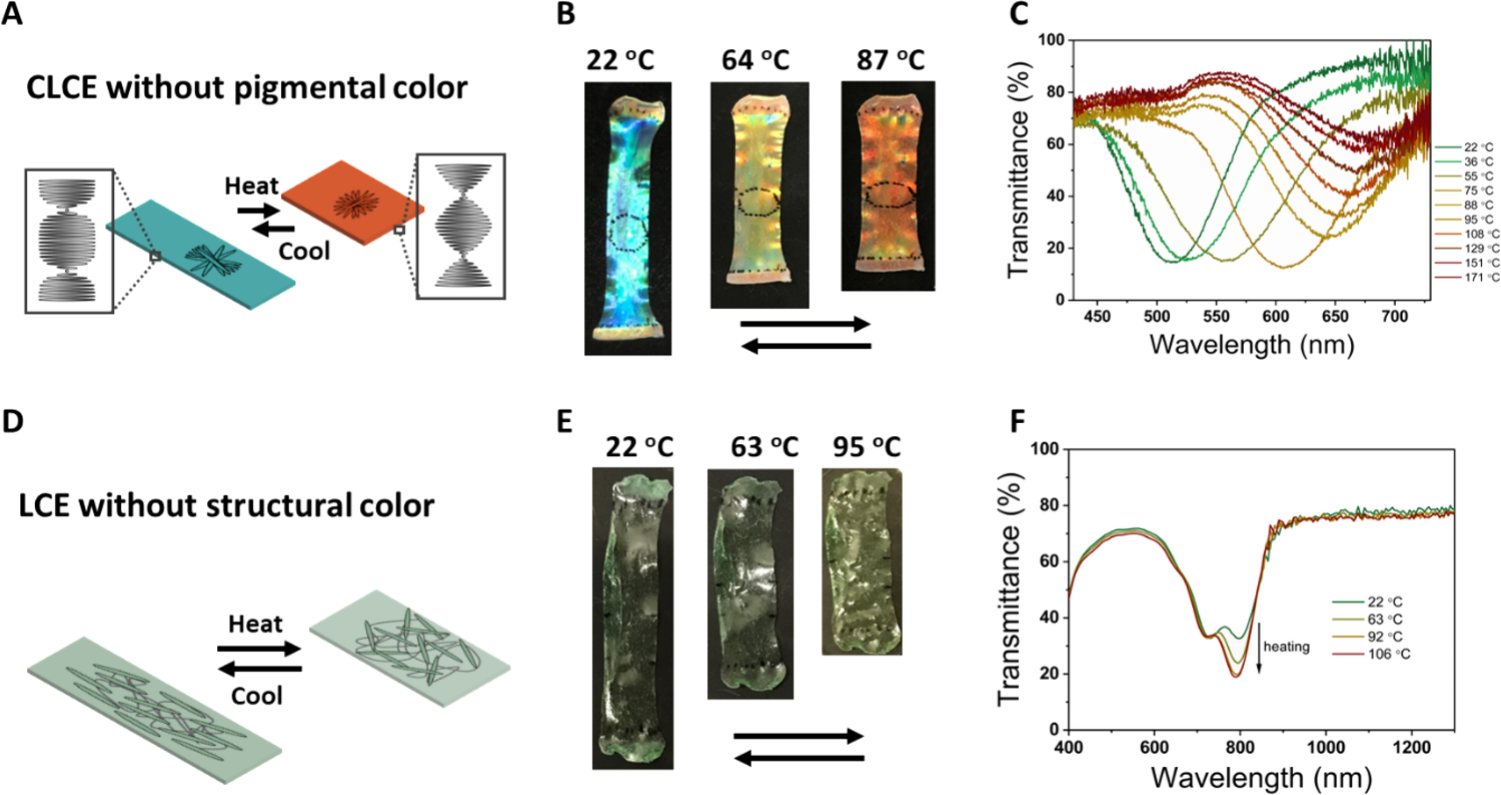
(A) Schematic illustration of reversible actuation and structural color change of the CLCE film. (B) Photographs and (C) transmittance spectra of a CLCE film without dye **9** at different temperatures. (D) Schematic illustration of reversible actuation, (E) photographs, and (F) transmittance spectra of a CLCE film prepared in the isotropic state at different temperatures.

To unravel the temperature-responsive reflection and absorption contribution, a CLCE film without the NIR dye and a LCE film without cholesteric order were prepared. To examine the temperature-responsive structural color, a CLCE film was prepared without the NIR dye using the same method as described in Section 2.1 (Figure 1B): the film after first cross-linking was uniaxially stretched to a strain of 112% and photopolymerized at room temperature. At 22 °C, the resulting film is greenish blue in color due to reflection, which is brighter compared to a pigmented CLCE, and shifts to red when heated to higher temperatures, which is reversible upon cooling (Figure 3A,B). The reflection band shifted from 513 to 654 nm upon heating from 22 to 95 °C (Figure 3C); further heating to 171 °C resulted in an additional reflection band shift of only 24 to 678 nm; these shifts are fully reversible upon cooling (Figure S4A,B). The length of the film decreased by 40% (from 35 to 21 mm), while the width increased by 27.1% (7 to 8.9 mm) upon heating from 22 to 102 °C (Figure S4C). These structural color and shape changes are similar to the pigmented structural colored actuators (Figure 2 versus Figure S4). As the film was uniaxially stretched and fully crosslinked, a deformed helix was expected to be present.^41,47^ This can be verified by measuring the transmittance spectra of circularly polarized light. Indeed, this film exhibits hyper-reflectivity with the same reflectivity at 510 nm when exposed to either right- (RCP) or left-handed circularly polarized (LCP) lights at 22 °C (Figure S4D, E), which is due to the helix deformation caused by uniaxial stretching, consistent with our previously reported work.^41^ Upon heating, the reflection intensity for both light polarizations drops (Figure S4F); the reflection of LCP drops more rapidly (from 70 to 15%) than the reflection of RCP (from 70 to 25%).

To determine the absorption behavior of the dye in the absence of reflection, a pigmented film without structural color was prepared. Using the same mixture for producing the film shown in Figure 2, thiol-acrylate Michael addition is carried out above the isotropic transition at 65 °C, resulting in polydomain formation and no planar cholesteric alignment (Figure 3D) and thus no structural color. The film appears light green due to the absorption of the dye (Figure 3E). This film shows similar actuation to the film with cholesteric alignment, with the length decreasing from 34 to 22.5 mm and the width increasing from 8 to 9.8 mm when heated from 22 to 103 °C (Figure S5A). Transmittance was measured at different temperatures, with two absorption peaks present at 725 and 795 nm (Figure 3F). Upon heating from 22 to 106 °C, the intensity of the peak at 725 nm remains constant, while the peak absorbance at 795 nm increases from 0.49 to 0.72; this absorbance change is fully reversible upon cooling (Figure S5B,C), although the color caused by the absorption of the dye does not show visible changes with temperature variation (Figure 3E). Thus, the polydomain film without a reflection band shows a similar absorption behavior to the aligned photonic film, indicating that the pigmentary color changes are independent from the structural color changes. This also shows that the CLCE film with a deformed helix behaves like a uniaxially aligned LC film, with similar shape changes.

### 2.3 Near-Infrared-Light-Fueled Pigmented 2D Structural Color Actuators

Light actuation of the CLCE is demonstrated using two different light sources: a diffuse halogen lamp that provides >90% of the light in the NIR region^48^ to demonstrate remote bulk actuation and an NIR (780 nm)-focused LED light used to show remote and local actuation. When the film is exposed to light from the halogen lamp, the film contracts and turns red with a temperature of 108 °C (Figure S6). It is noted that some regions of the film appear to remain green with the light on, which is attributed to the film not being totally flat, causing viewing angle differences and an effective blueshift of the perceived color. It is worth mentioning that the halogen lamp also induces heating and actuation for films without added NIR dye (Figure S7) as the light irradiation can generate enough heat to warm the sample despite the absence of dye.

**Figure 4:**
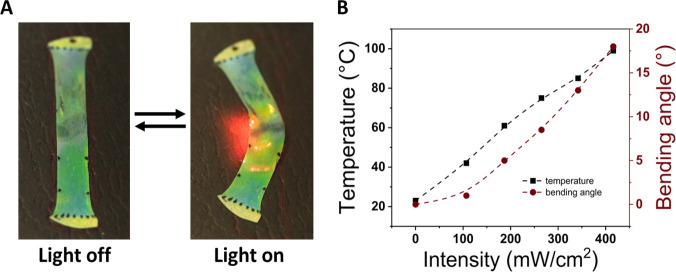
(A) Photographs of the CLCE film showing in-plane bending generated by locally illuminating the film with 780 nm NIR light (416 mW/cm^2^). (B) Temperature and the bending angle as a function of the intensity of the illumination light (dashed lines are plotted to guide the eye).

Interestingly, when locally illuminating with 780 nm NIR light, a film lying on a sheet of black paper shows an in-plane bending motion (Figure 4A and Figure S8). Hanging the same film in air and exposing it to 780 nm NIR light also induces in-plane bending (Figure S9 and Videos S2 and S3; Video S2 shows the reversibility of the bending after a second exposure: samples were cycled at least 10 times with no obvious change in actuation). The color of the unexposed area film is not changing, indicating that this region is not heating up. IR camera measurements also reveal local heating (Figure S9): the temperature of the exposed region reaches 65 °C, while the unexposed regions remain at 22 °C. The temperature of the rear side of the film is also measured, and there is no apparent temperature difference between the front and rear sides. Increasing the intensity of the light source increases the temperature and the bending angle of the film accordingly (Figure 4B). The higher intensity results in a stronger photothermal effect via the dye; thus, a higher local temperature and steeper thermal gradient between the exposed and unexposed regions are achieved. It is easy to adjust the degree of bending by changing the light intensity. To demonstrate the necessity of using dye to achieve light-fueled local actuation in the CLCE films with the NIR light, a film containing no IR 788 was locally exposed to the NIR light, but no obvious in-plane bending was observed, either when lying on the table with a sheet of black paper as a background or when hanging in air (Figure S10 and Video S4). This control experiment demonstrates that the photothermal effect of IR 788 dye transforming light into heat is vital in achieving light-driven locally controlled actuation.

The uniaxial stretching process is critical in realizing the deformed helix and corresponding uniaxial-like actuation in the CLCE film, which is necessary to achieve in-plane bending in the CLCE film, an actuation not possible in a classic cholesteric system. To verify this mechanism, a CLCE film photo-cross-linked at 0% strain is prepared (Figure S11). When exposed to 780 nm NIR light, the maximum temperature measured in the exposed area is 75 °C, while the unexposed area remains at 22 °C. The film did not show any obvious in-plane bending even with this considerable temperature difference because the actuation of a non-deformed CLCE film is very small, less than 10%,^41^ compared to a stretched film, which is up to 35%. This observation indicates that the in-plane bending is unique to the stretched CLCE film, meaning that stretching and creation of a deformed helix are essential in achieving in-plane bending motion in a cholesteric film due to local photothermal contraction along the stretching direction when locally exposed to light.

### 2.4 Near-Infrared-Light-Fueled Pigmented 3D Structural Color Actuators

**Figure 5:**
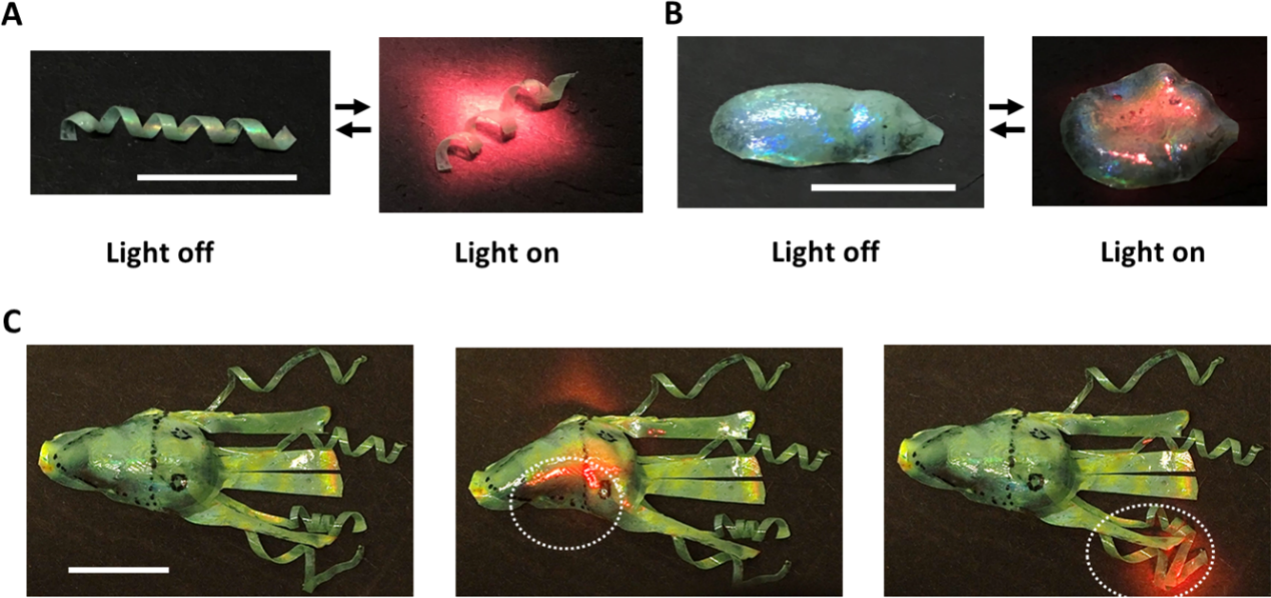
Photographs of (A) 3D spiral-shaped and (B) 3D cone-shaped CLCE with 780 nm NIR light off and on. (C) Photographs of the 3D shaped “cuttlefish” made from CLCE with the light off and when the head and the spiral arms are exposed to 780 nm NIR light. Scale bars = 10 mm.

CLCE films are not restricted to being flat: 3D spirals and cone-shaped films can also be created. To make the spiral-shaped CLCE, after the thiol-acrylate Michael addition, the film was stretched with a strain of around 60% and wrapped around a 2 mm diameter cylindrical mold (Figure 1B). The CLCE film was exposed to UV light to fully cross-link the film and fix the programmed spiral shape. The resulting CLCE film has a spiral shape and a green color (Figure 5A). When the halogen light is on, the spiral turns red and unwinds as it returns to its original state as the sample reaches a temperature of 95 °C (Figure S12A,B). With the 780 nm NIR LED light, local unwinding is achieved (Figure 5A and Video S5) as a result of a local maximum temperature of 72 °C (Figure S12C).

The cone-shaped CLCE was made by first stretching the partially cross-linked CLCE film to a strain of 42%, pressing the film between a positive and negative cone-shaped mold, and full cross-linking using UV light (Figure 1B). The cone-shaped CLCE is green at 22 °C, and, when the halogen light is on, the temperature of the CLCE increased to 111 °C with a concurrent color change to red and loss of its cone shape (Figure S12D,E). Local actuation was demonstrated using 780 nm NIR LED light: when the top region of the cone was exposed to NIR light, local actuation is observed, i.e., only the part exposed to light shows flattening (Figure 5B and Videos S6 and S7) with a local temperature of 102 °C (Figure S12F).

A 3D “cuttlefish” was prepared by combining the flat spiral and cone-shaped CLCE elements (Figure 5C). The body of the cuttlefish was molded with a cone shape using the method described above. The edge of this molded CLCE, which was stretched, was trimmed to form four straight “arms”. The spiral CLCE arms are affixed to the cone-shaped body using adhesive tape. The cuttlefish is initially green at 22 °C. When the halogen lamp is turned on, the spiral arms unwind, the straight arms contract and change to red color, and the body flattens and turns red, with the temperature reaching 134 °C (Figure S13). More local control of the cuttlefish is demonstrated using a 780 nm NIR light (Figure 5C and Video S8): when the “head” of the cuttlefish is exposed to the NIR light, it senses the light immediately and starts to contract and modify its color locally. When the spiral arms are exposed to light, the cuttlefish reacts by unwinding the tips of the arms. This cuttlefish demonstrates the possibility of environmental sensing and response by changing its appearance, holding promise for use as camouflage in soft robotics.

## 3 Conclusions

Pigmented structural color actuators inspired by cuttlefish capable of changing their overall appearance for camouflage by altering both body shapes and colors (both structural and pigmentary colors) are successfully created. The temperature-dependent aggregation and de-aggregation of a dye reported here might be a new way to create pigmented color changes in a polymer film. The actuation and color changes can be driven by light using the photothermal effect of the embedded NIR absorbing dye. On the one hand, cooperative tunable structural color and absorption properties are successfully achieved, mimicking the color-changing ability of the cuttlefish. On the other hand, in-plane bending motion using NIR light is achieved in the flat CLCE film. 3D-shaped objects, including spiral and cone shapes, are also demonstrated, showing the possibility of using light to locally trigger both actuation and color changes. Finally, a 3D-shaped cuttlefish demonstrator that can sense light and respond by changing its appearance locally was demonstrated. The pigmented structural color actuators might be interesting in soft robotics for camouflage and signaling, for example. The future research direction lies in the development of pigmented structural color actuator assemblies capable of moving, sensing, and adapting.

## 4 Materials and Methods

### 4.1 Chemicals

Diacrylate liquid crystal monomers **1** and **2** were purchased from Merck. Monomer **3** and Lumogen IR 788 were received from BASF. 2,2′-(Ethylenedioxy) diethanethiol (**4**), pentaerythritol tetrakis(3-mercaptopropionate) (**5**), dipropylamine (**6**), inhibitor 4-methoxyphenol (MEHQ), and polyvinyl alcohol (PVA) were obtained from Sigma-Aldrich. The vinyl cross-linker (**7**) was obtained from Sigma-Aldrich. Photoinitiator **8** was obtained from CIBA. Photothermal dye (**9**) was obtained from BASF. All reagents were used as received, without further purification.

### 4.2 PVA-Functionalized Glass Substrate

To prepare the PVA functionalized glass substrate, 3 × 3 cm^2^ glass plates were cleaned in acetone for 30 min using ultra-sonication followed by 30 min in ethanol and subsequently treated by UV–ozone (PR-100, Ultra Violet Products) for 20 min. Then, 5 wt % PVA with a molecular weight of 9000 was dissolved in distilled water and spin-coated on a clean 3 × 3 cm^2^ glass plate using a spin coater (Karl Suss CT 62) by rotating at 2500 rpm for 30 s. The PVA-coated glass plates were then placed at 60 °C for 30 min to evaporate the water.

### 4.3 Preparation of CLCE Films

Here, 46.21 wt % (1077.0 mg, 1.6 mmol) monomer **1**, 17.32 wt % (403.7 mg, 0.686 mmol) monomer **2**, 3.95 wt % (92.0 mg, 0.095 mmol) chiral dopant **3**, 17.47 wt % (407.0 mg, 2.232 mmol) dithiol **4**, 7.8 wt % (181.8 mg, 0.372 mmol) tetrathiol **5**, 4.25 wt % (99.0 mg, 0.397 mmol) vinyl cross-linker **7**, 1 wt % (23.3 mg) photoinitiator **8**, and 0.5 wt % (11.6 mg) 4-methoxyphenol were added to a vial. Four milliliters of dichloromethane (DCM) was added to the vial to dissolve the mixture and ensure good mixing. The vial was magnetically stirred on an 80 °C hotplate around 1.5 h to remove the DCM. Approximately 400 mg of the CLC mixture was added to a small vial, 0.5 wt % catalyst **6** was gently added, and the vial was gently heated using a heat-gun and mixed on the vortex mixer to ensure that the catalyst is properly blended with the CLC mixture. It is important that this step must be very gentle and rapid as the catalyst can initiate the first-step thiol-Michael addition quickly, causing local cross-linking and a non-uniform film with poor alignment, which is not desired. Around 120 mg of this mixture was placed between PVA-coated glass plates with pieces of Scotch tape glued onto the edges to serve as spacers (thickness around 130 μm), forming a cell. The filled cell was placed on a 15 °C hotplate and sheared to achieve the proper alignment: the cell remained at 15 °C for 1 h and 22 °C for another hour to finish the thiol-Michael addition. The cell was maintained below room temperature to ensure that the CLC mixture is in the cholesteric phase as the cholesteric phase is essential to obtain the planer alignment and structural color. The cell was then immersed in water at 50 °C for around 2 h to open the cell, releasing the film from the glass plates and obtaining a freestanding film. The film was uniaxially stretched at a specific strain to program the desired shape and color and photo-polymerized in a nitrogen box with UV light using an Omnicure S2000 UV lamp (300–400 nm) at an intensity of 20 mW cm^–2^ for 10 min on one side; the film was then flipped over and exposed for another 10 min to ensure uniform cross-linking on both sides of the film. For the light-driven film, 0.07 wt % IR 788 was added in the monomer mixture and the same method was used to prepare the film.

### 4.4 Preparation of 3D-Shaped Cuttlefish

To make the spiral shape, the film after thiol-acrylate polymerization was first stretched and then wrapped around a cylindrical mold with a diameter of 2 mm. The CLCEs were then exposed to UV light for 20 min to fully cross-link the films and fix the programmed spiral shape. To make the cone-shaped body, the film after thiol-acrylate polymerization was first stretched and then pressed between positive and negative cone-shaped molds and then fully cross-linked for 20 min. This molded CLCE was then trimmed to form four straight arms. Finally, the spiral arms were glued with the cone-shaped body using adhesive tapes to make a complete cuttlefish.

### 4.5 Characterization

Differential scanning calorimetry (DSC) curves were measured with a DSC Q1000 from TA Instruments. A rate of 10 °C min^–1^ was used for both heating and cooling ramps. The transmittance spectra for the film with no dye shown in Figure 3C and Figure S4 were measured using an Ocean Optics spectrometer attached to a polarized optical microscope with 20× objective. The transmittance of the film with dye IR 788 shown in Figure 2C and Figure S3 was measured using a Perkin Elmer Lambda 750 UV/Vis/NIR spectrophotometer. The temperature of the film was controlled by a Linkam THMS600 hot stage. The films were placed on a hotplate, and the lengths and widths of the films were measured at different temperatures to determine the thermal actuation. Photographs and videos were taken with a Sony Cyber-shot camera and an iPhone 7. Local NIR light illumination was performed with an LED light source emitting 780 nm (ThorLabs M780L3) mounted with a collimator (ThorLabs SM2F32-1) and driven by a controller (ThorLabs). The intensity was 416 mW/cm^2^ unless indicated. The distance between the LED source and the sample was around 10 cm. The NIR (780 nm) LED light spectra were measured with an integrating sphere (Labsphere LMS-100), and the intensity was calculated from the integration of the spectra (Figure S14). The bulk NIR light illumination was performed with a halogen lamp (FlashTorch, Wicked Lasers), which provided more than 90% of the light in the NIR region.^48^ The infrared thermal imaging device (Ti32 Infrared Camera, Fluke) was used to detect the temperature distribution of the film when exposed to the NIR light source. The stress–strain curves were performed on samples (partially cross-linked CLCE: 5.5 × 7.2 × 0.126 mm^3^, fully cross-linked CLCE: 7.6 × 6.0 × 0.098 mm^3^) at 25 °C with a TA Instruments Q800 in vertical tension mode at an elongation rate of 2 mm/min.

## Supporting Information Available

Reversible temperature response (22–110 °C) of the CLCE film with dye shown in Figure 2 (MP4)
780 nm NIR light response of the CLCE film with dye shown in Figure 2 (front view) (MP4)
780 nm NIR light response of the CLCE film with dye shown in Figure 2 (side view) (MP4)
780 nm NIR light response of the CLCE film without dye shown in Figure 3B (MP4)
780 nm NIR light response of the spiral CLCE shown in Figure 5A (MP4)
780 nm NIR light response of the cone CLCE shown in Figure 5B (top view) (MP4)
DSC curves, stress–strain curves, optical properties and actuation of the CLCE film with no dye, optical properties of the CLCE film with dye, optical properties and actuation of the film with no structural color, halogen lamp and 780 nm NIR light actuation and IR images of the CLCE 2D films and 3D objects, and NIR light intensity measurement (PDF)
780 nm NIR light response of the cone CLCE shown in Figure 5B (side view) (MP4)
780 nm NIR light response of the cuttlefish shown in Figure 5C (MP4)
