# Supplementary material for: Pigmented Structural Color Actuators Fueled by Near-Infrared Light
Source: ACS Appl Mater Interfaces. 2022 Apr 22;14(17):20093–100. doi: 10.1021/acsami.2c03392 (PMC9073939; doi:10.1021/acsami.2c03392)
Supplement: Supplementary file 7 — am2c03392_si_007.pdf [file am2c03392_si_007.pdf]

Supporting Information

Pigmented structural color actuators fueled by near infrared light

Pei Zhang[1], Michael G. Debije[1], Laurens T. de Haan,[2*] and Albert P. H. J. Schenning[1*]

[1] Stimuli-responsive Functional Materials and Devices, Department of Chemical Engineering and Chemistry, Eindhoven University of Technology, P.O. Box 513, 5600 MB Eindhoven, The Netherlands.

Email: a.p.h.j.schenning@tue.nl

[2] SCNU-TUE Joint Lab of Device Integrated Responsive Materials (DIRM), National Center for International Research on Green Optoelectronics, South China Normal University, Guangzhou, 510006, P. R. China.

Email: ldhaan@m.scnu.edu.cn

Video S1. Reversible temperature response (22 ^{o}C to 110 ^{o}C) of the CLCE film with dye shown in Figure 2.

Video S2. 780 nm NIR light response of the CLCE film with dye shown in Figure 2, front view.

Video S3. 780 nm NIR light response of the CLCE film with dye shown in Figure 2, side view.

Video S4. 780 nm NIR light response of the CLCE film without dye shown in Figure 3B.

Video S5. 780 nm NIR light response of the spiral CLCE shown in Figure 5A.

Video S6. 780 nm NIR light response of the cone CLCE shown in Figure 5B, top view.

Video S7. 780 nm NIR light response of the cone CLCE shown in Figure 5B, side view.

Video S8. 780 nm NIR light response of the cuttlefish shown in Figure 5C.

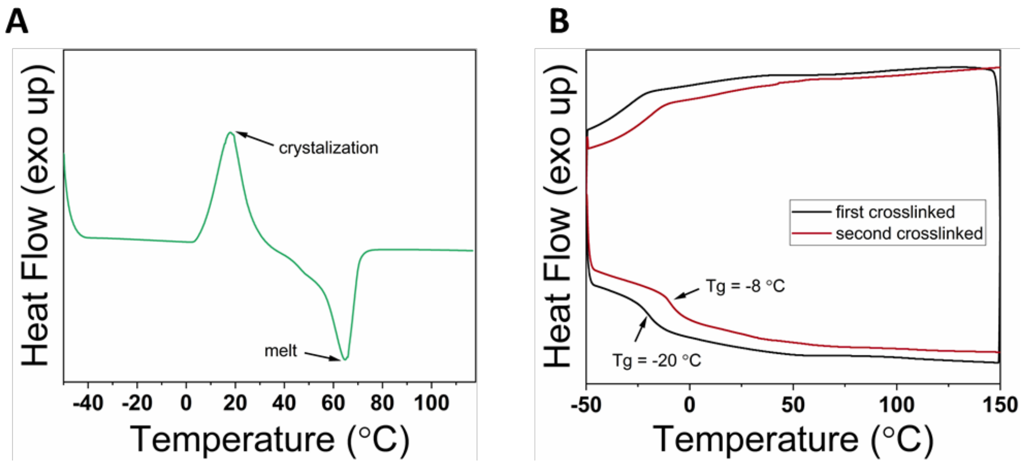

Figure S1. (A) Differential scanning calorimetry (DSC) curve of the CLC monomer mixture with dye **9** and without catalyst before thiol-acrylate Michael addition. (B) DSC curves of the CLCE film with dye **9** after the first crosslinking (thiol-acrylate Michael addition) and second crosslinking (radical photopolymerization) steps.

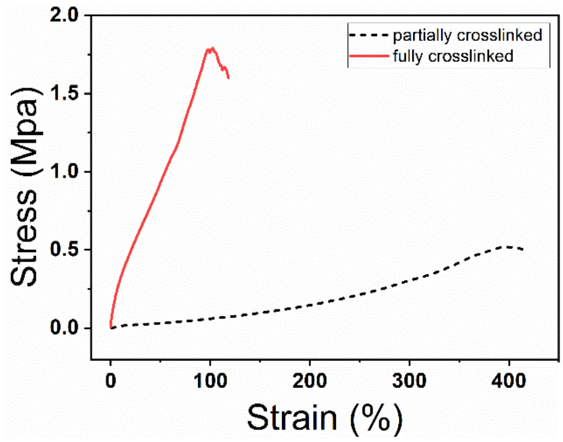

Figure S2. Stress-strain curves of the CLCE with dye **9** after the first crosslinking (black dashed line) and second crosslinking (red solid line) steps.

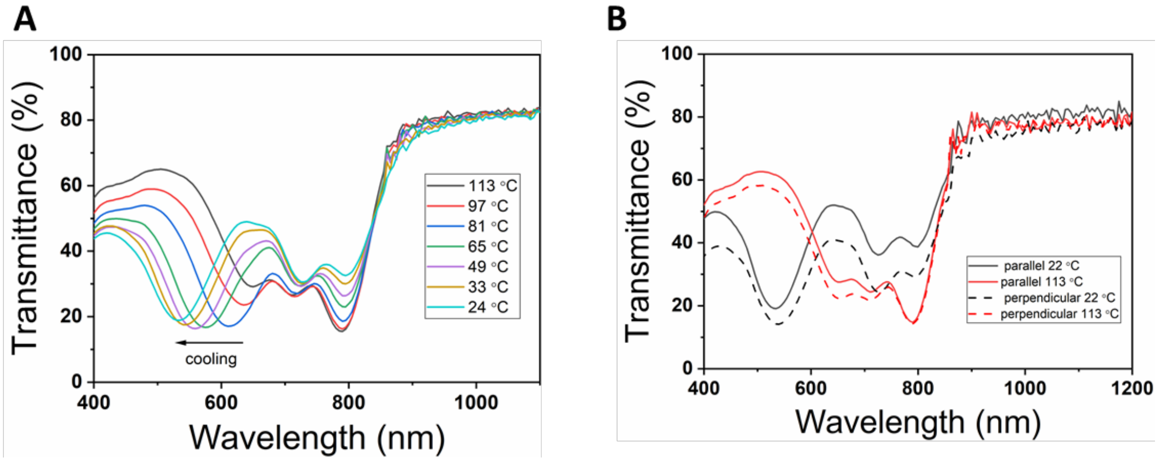

Figure S3. (A) The transmittance spectra of the CLCE film with dye **9** shown in Figure 2 measured at different temperatures upon cooling. (B) The transmittance spectra of the film CLCE film with dye **9** shown in Figure 2 measured with linearly polarized light at different temperatures with the stretching direction of the film parallel and perpendicular to the direction of the polarized light.

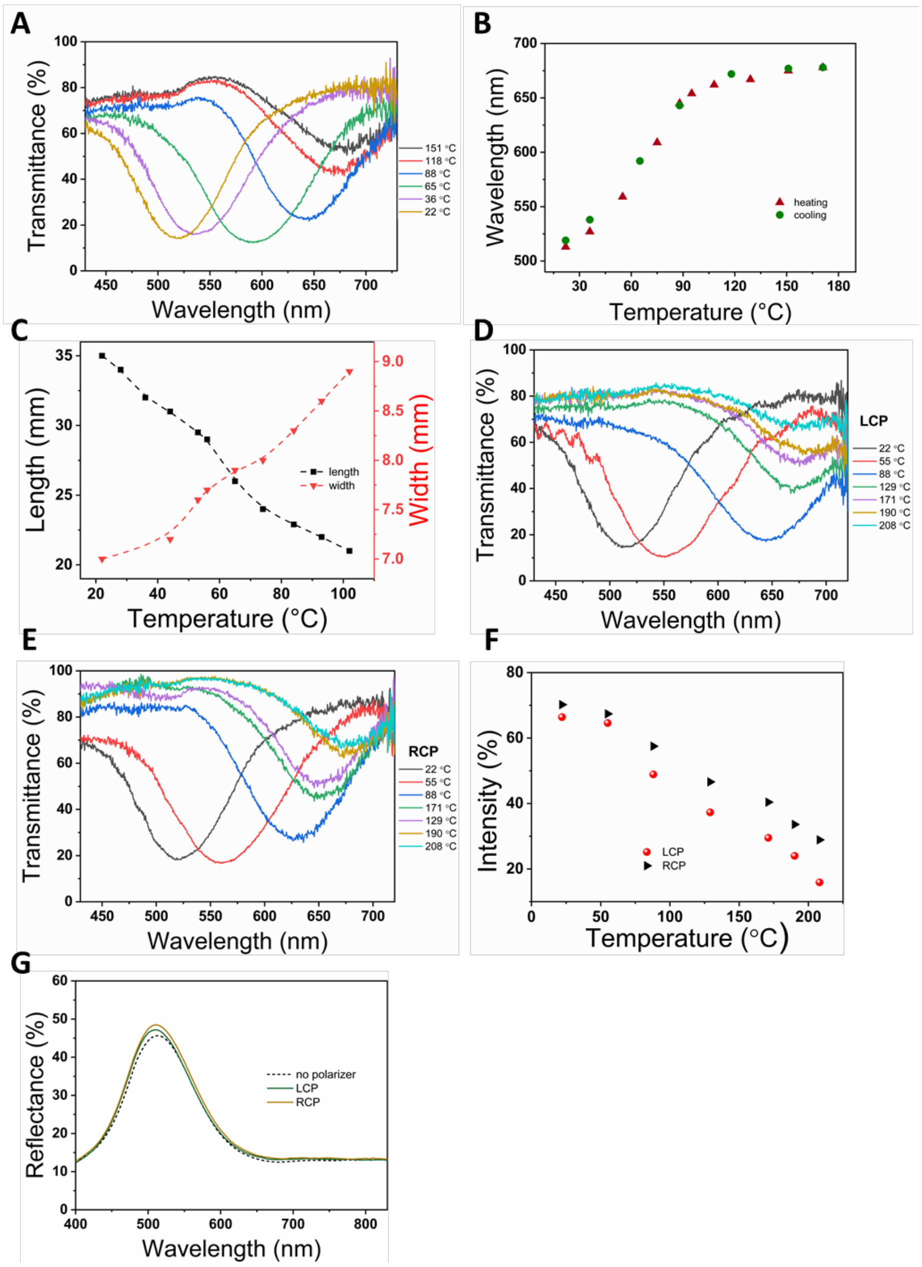

Figure S4. The optical properties and actuation of the film shown in Figure 3B. (A) The transmittance spectra of the CLCE film measured without dye **9** at different temperatures upon cooling. (B) The reflection wavelength as a function of temperature upon heating and cooling. (C) The length (the stretching direction) and width (perpendicular to the stretching direction) of the film as a function of temperature (dashed lines are plotted to guide the eye). The transmittance spectra of the CLCE film measured with (D) left-handed (LCP) and (E) right-handed circularly polarized light (RCP). (F) The reflection intensity measured with LCP and RCP as a function of temperature. (G) The reflectance spectra of the CLCE film without dye **9** measured at 22 °C with unpolarized light and with LCP/RCP.

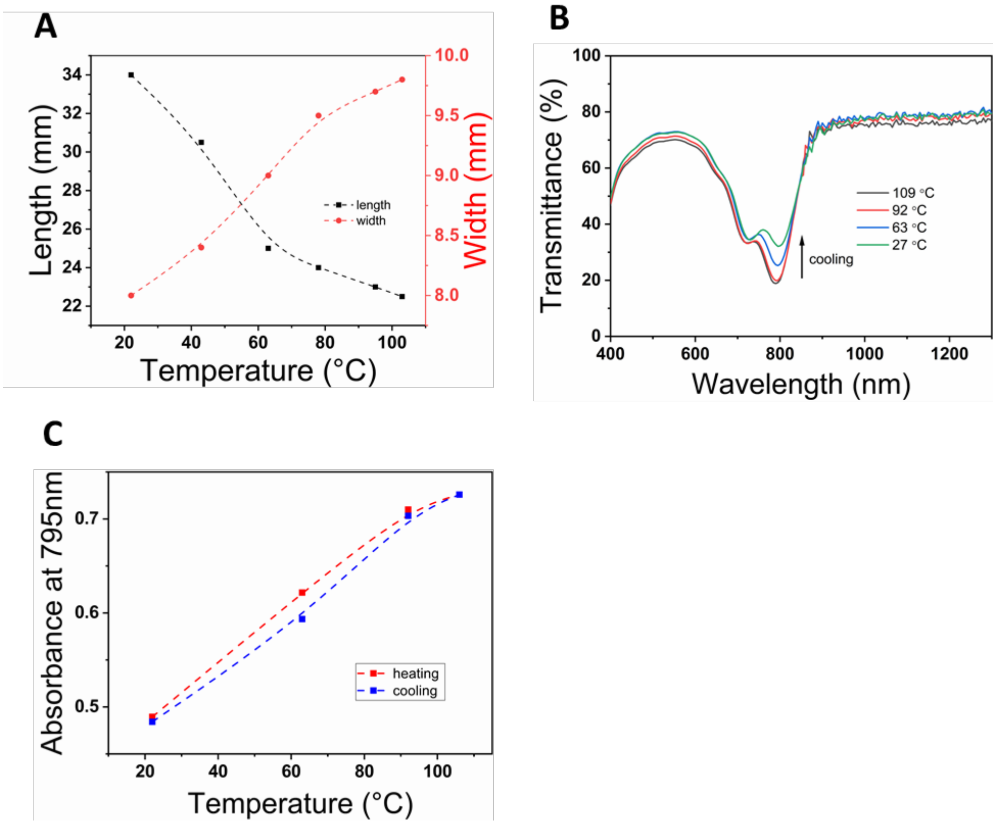

Figure S5. (A) The length (the stretching direction) and width (perpendicular to the stretching direction) of the CLCE film prepared in the isotropic state as a function of temperature (dashed lines are plotted to guide the eye). (B) Transmittance spectra of the film at different temperatures upon cooling. (C) The absorbance at 795 nm as a function of temperature upon heating and cooling (dashed lines are plotted to guide the eye).

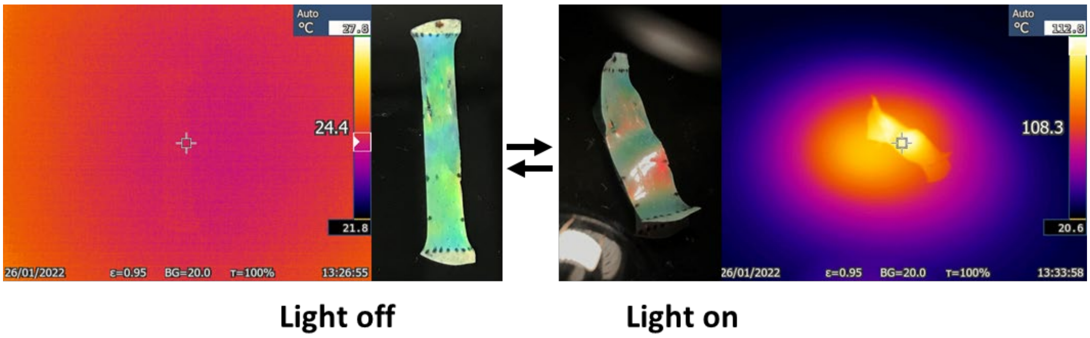

Figure S6. Photographs and IR images of the CLCE film with dye **9** shown in Figure 2 lying on the table when halogen lamp off (left images) and on (right images).

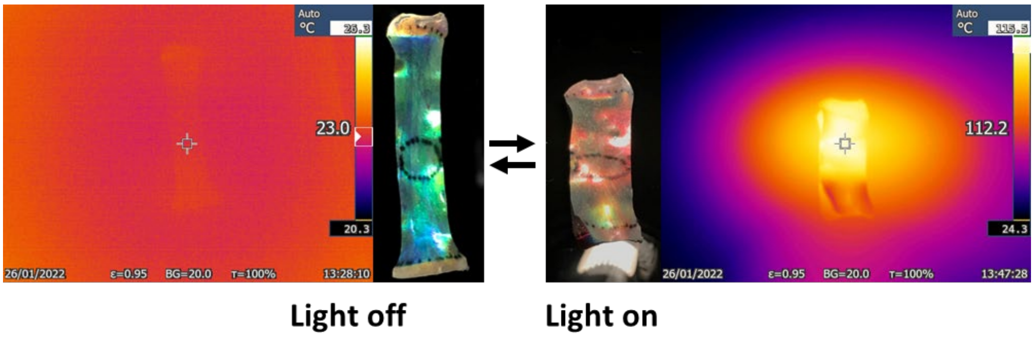

Figure S7. Photographs and IR images of the film without dye **9** shown in Figure 3B lying on the table when halogen lamp off (left images) and on (right images).

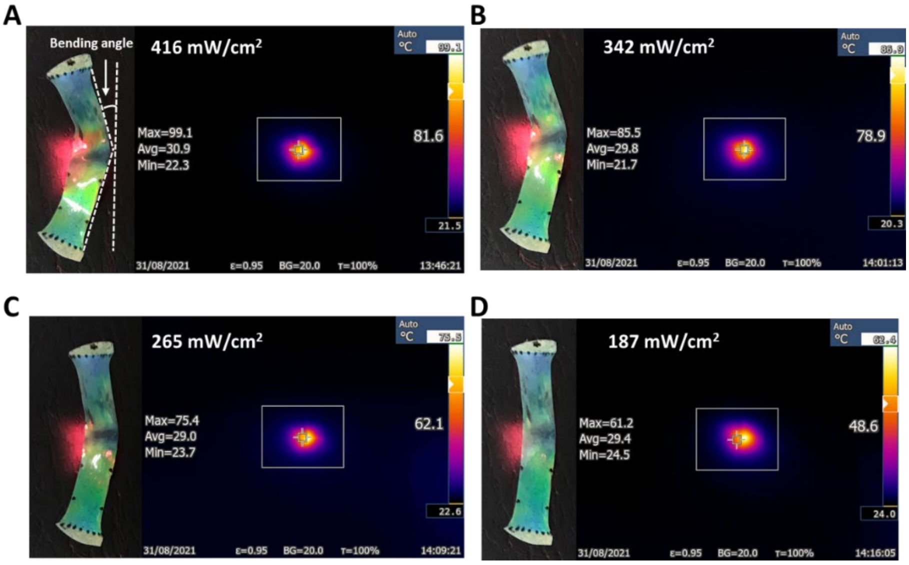

Figure S8. Photographs and IR images of the CLCE film shown in Figure 2 when exposed to 780 nm NIR light at different intensities.

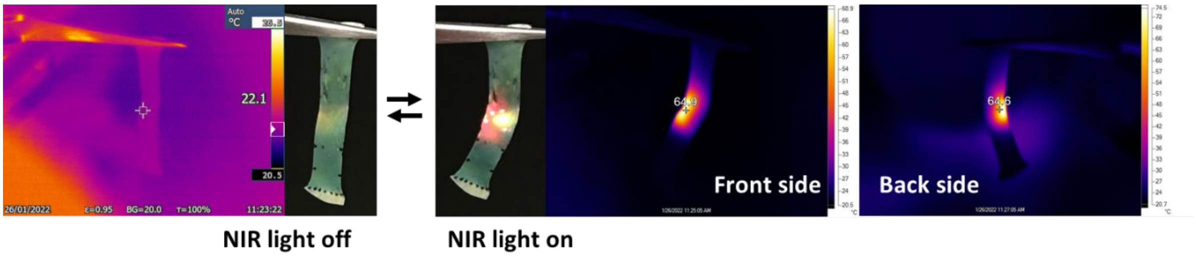

Figure S9. Photographs and IR images of the CLCE film shown in Figure 2 hanging in the air with 780nm NIR light off (left) and on (right; IR images from both front and back sides). NIR light intensity: 416 mW/cm^2.

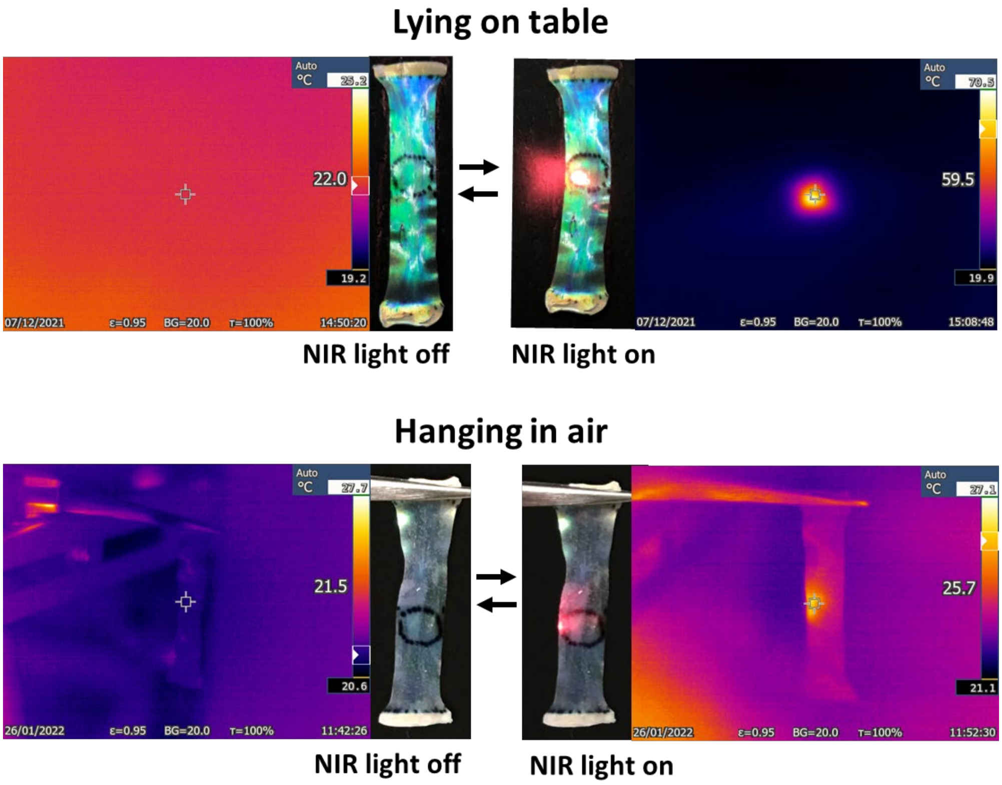

Figure S10. Photographs and IR images of the CLCE film without dye **9** placed on the table with a black paper as background (top row) and when hanging in the air (bottom row) with 780nm NIR light off (left) and on (right) (intensity 416 mW/cm^2). When hanging in the air, the temperature of the light exposed region of the film was measured as 25.7 ^{o}C with no obvious in-plane bending. When lying on the table, the exposed area was measured as around 59 ^{o}C, likely due to the black paper background absorbing light and heating up, also explaining why the temperature of the film containing dye (shown in Figure 2) is higher when lying on a black surface compared to hanging in the air (100 ^{o}C vs. 65 ^{o}C) when exposed to the same light intensity.

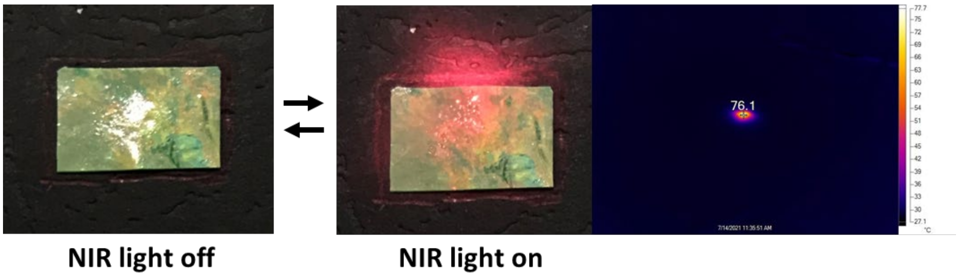

Figure S11. Photographs of the CLCE film with dye **9** that was photo-crosslinked with a strain of 0% with 780nm NIR light off (left) and on (right). NIR light intensity: 416 mW/cm^2.

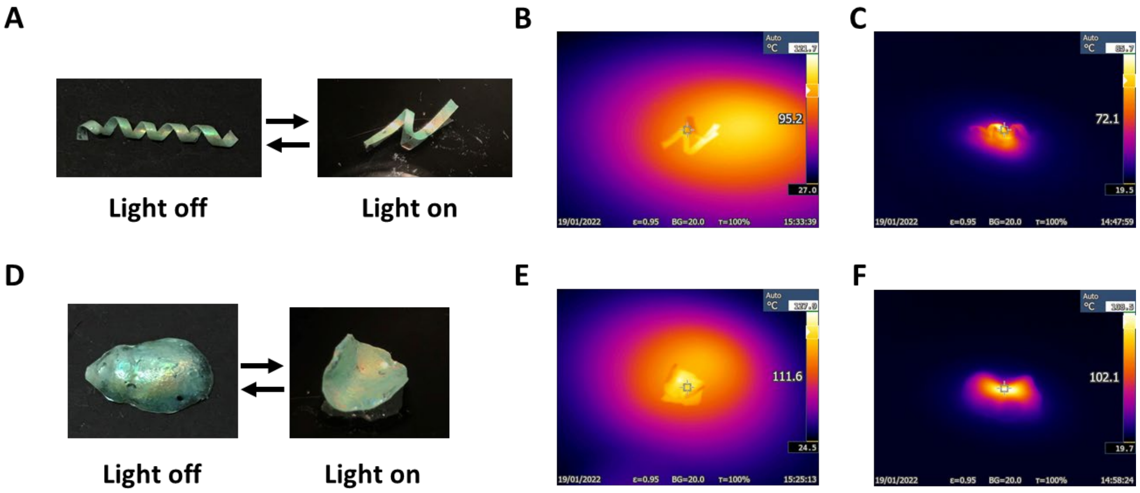

Figure S12. (A) Photographs and (B) IR image of 3D spiral shaped CLCE film with halogen lamp on. (C) IR image of 3D spiral shaped CLCE film with 780nm NIR light on. (D) Photographs and (E) IR image of 3D cone shaped CLCE film with halogen lamp on. (F) IR image of 3D cone shaped CLCE with 780nm NIR light on.

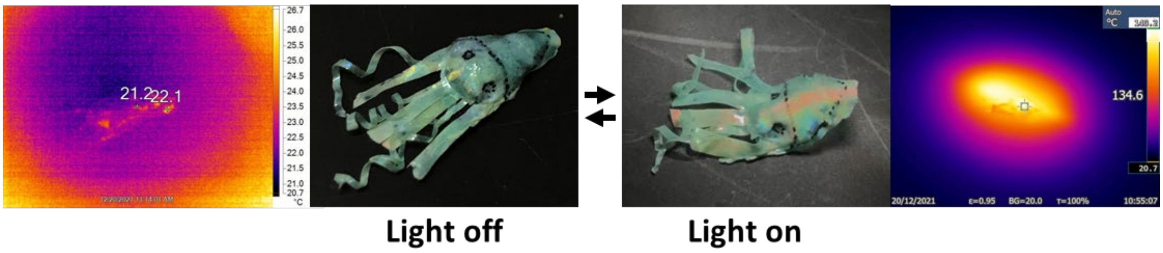

Figure S13. Photographs and IR images of the CLCE cuttlefish with halogen lamp off (left images) and on (right images).

A

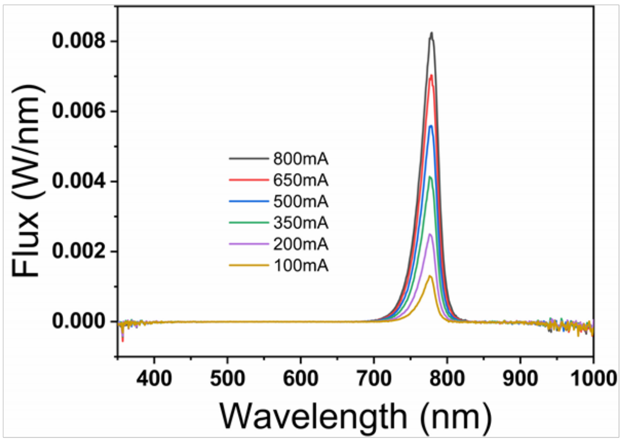

B

Current/mA	Integration/W	mW/cm²
800	0.26643	416.2969
650	0.21902	342.2188
500	0.16965	265.0781
350	0.11979	187.1719
200	0.06869	107.3281
100	0.03336	52.125

$$y = 0.5203x + 2.9007$$

Figure S14. 780 nm NIR LED light intensity measurement. (A) Spectra measured using an integrating sphere. (B) The integrations of the spectra and calculated light intensities with different controller currents. The light spot has an area of 0.64 cm^2. The relation between the current of the controller x (mA) and the light intensity y (mW/cm^2) is fitted and the resulting equation is shown.
